# An Electrochemical Microsensor Based on a AuNPs-Modified Microband Array Electrode for Phosphate Determination in Fresh Water Samples ^†^

**DOI:** 10.3390/s141224472

**Published:** 2014-12-19

**Authors:** Fangfang Wang, Jianhua Tong, Yang Li, Chao Bian, Jizhou Sun, Shanhong Xia

**Affiliations:** 1 State Key Laboratory of Transducer Technology, Institute of Electronics, Chinese Academy of Sciences, Beijing 100190, China; E-Mails: wffang_89@163.com (F.W.); math101@163.com (Y.L.); cbian@mail.ie.ac.cn (C.B.); sunjizhou628@163.com (J.S.); shxia@mail.ie.ac.cn (S.X.); 2 University of Chinese Academy of Sciences, Beijing 100190, China

**Keywords:** electrochemical, microband array electrode, gold nanoparticles, phosphate, fresh water samples

## Abstract

This work describes the fabrication, characterization, and application of a gold microband array electrode (MAE) for the determination of phosphate in fresh water samples. The working principle of this MAE is based on the reduction of a molybdophosphate complex using the linear sweep voltammetric (LSV) method. The calibration of this microsensor was performed with standard phosphate solutions prepared with KH_2_PO_4_ and pH adjusted to 1.0. The microsensor consists of a platinum counter electrode, a gold MAE as working electrode, and an Ag/AgCl electrode as reference electrode. The microelectrode chips were fabricated by the Micro Electro-Mechanical System (MEMS) technique. To improve the sensitivity, gold nanoparticles (AuNPs) were electrodeposited on the working electrode. With a linear range from 0.02 to 0.50 mg P/L, the sensitivity of the unmodified microsensor is 2.40 μA per (mg P/L) (*R*^2^ = 0.99) and that of the AuNPs-modified microsensor is 7.66 μA per (mg P/L) (*R*^2^ = 0.99). The experimental results showed that AuNPs-modified microelectrode had better sensitivity and a larger current response than the unmodified microelectrode.

## 1. Introduction

Phosphorus is an essential element for organisms in the aqueous environment. Total phosphorus (TP), defined as the sum of orthophosphate, condensed phosphates (pyro-, meta- and polyphosphates) and organically bound phosphorus, plays a key role in the water environment. Excessive TP causes the phenomenon of eutrophication and algae bloom bursts, and reduces dissolved oxygen in lakes and reservoirs [1].

Most TP determination methods include two steps: the first step is a digestion process which turns all forms of phosphorus into orthophosphate, and the second step is the detection process which obtains the concentration of TP by detecting the concentration of orthophosphate in the digested samples. The common techniques of determination of orthophosphate mainly include optical methods [2,3] and electrochemical methods [4]. Optical methods, such as spectrophotometric and chromatographic methods, involve the treatment of the sample with an acidic molybdate solution to produce a phosphomolybdate complex which is further reduced by ascorbic acid to give an intensely colored product. They are prone to interference, unstable or erratic measurements and a lack of selectivity due to the fact that some reagents can produce emission with more than one analyte [5]. Electrochemical methods provide a promising approach to decrease the response time and have less interference from dissolved silicon or turbidity. Additionally, electrochemistry also has the advantages of long lifetime, high precision, low detection limit and good reproducibility. Electrochemical phosphate sensors include biosensors (such as enzyme electrode [6,7] and plant tissue electrodes [8–10]), ion-selective electrodes [11,12] and metal electrodes [13,14]. The determination of phosphate has been performed by voltammetry with carbon paste electrodes [15], gold microdisk electrodes [16], and glassy carbon electrodes [17], and amperometric procedures are also reported [18,19]. Several studies on gold electrodes based on molybdophosphate complex with an electrochemical method for phosphate detection have already been published, however, the current response is very low under the selected voltage using the chronoamperometry method [20–22].

Nanomaterials have been extensively investigated in electrochemistry for sensor fabrication, due to their small size effects, surface effects and quantum tunneling effects. Gold nanoparticles (AuNPs) have good biocompatibility. AuNPs can be prepared by chemical synthesis or electrodeposition techniques. Electrodeposition is rapid, cheap and easily controllable method. The modes of electrodeposition mainly include chronoamperometry [23] and cyclic voltammetry [24]. Chronoamperometry has the advantage of controlling the morphology and the density of the resulting AuNPs [25].

In this work, we use a three-electrode-system to detect phosphate using linear sweep voltammetry. A gold MAE is developed as working electrode by the MEMS technique and AuNPs were electrodeposited on this gold MAE. SEM images and cyclic voltammtry responses in sulphuric acid were determined for the unmodified and AuNPs modified gold microelectrode. The working principle of this MAE is based on the reduction of the molybdophosphate complex using a linear sweep voltammetric method. The current of the second reduction peak was selected to record the phosphate response. From the experimental results, we can see that the AuNPs-modified microsensor has a good linearity for phosphate. The AuNPs-modified gold microelectrode provides a much larger current response and better sensitivity than the corresponding unmodified gold microelectrode. The characteristics of this AuNPs-modified microsensor could meet the requirements for surface water monitoring and classification.

## 2. Experimental Section

### 2.1. Reagents and Equipments

Phosphate standard solutions were prepared with potassium dihydrogen phosphate (KH_2_PO_4_) (Sinopharm Chemical Reagent, Beijing, China) diluted to different concentrations using ultra-pure water. The molybdate sodium solution (20 mM) was prepared with molybdate sodium (Sinopharm Chemical Reagent, Beijing, China) (Na_2_MoO_4_) dissolved in ultra-pure water. The detected solutions were mixtures of phosphate standard solution and molybdate sodium solutions in a volumetric proportion of 1:1. Gold deposition solution (2 mM) was prepared with chloroauric acid (Sinopharm Chemical Reagent, Beijing, China) (HAuCl_4_) dissolved in 0.5 mol/L sulphuric acid (Sinopharm Chemical Reagent, Beijing, China) (H_2_SO_4_). The oxidant of digestion was 40 g/L potassium persulfate (Sinopharm Chemical Reagent, Beijing, China) (K_2_S_2_O_4_) which was dissolved in ultra-pure water and kept in a brown glass bottle. All the detected solutions were adjusted to pH = 1.0 with 98% sulfuric acid (Sinopharm Chemical Reagent, Beijing, China), and kept in polypropylene bottles. All reagents used were of analytical grade. Calibration and measurements of the sensor were carried out in a 15 mL polypropylene bottle with a three-electrode-system which consists of a platinum plate as counter electrode, a gold MAE as working electrode, and an Ag/AgCl electrode as reference electrode. The electrodeposition of AuNPs on the gold microband array electrode and all the electrical signal generation and detection processes were carried out in the gold deposition solution using a three-electrode system with a Reference-600 electrochemical analyser (Gamry Instruments, PA, USA).

### 2.2. Electrode Fabrication

**Figure 1.**
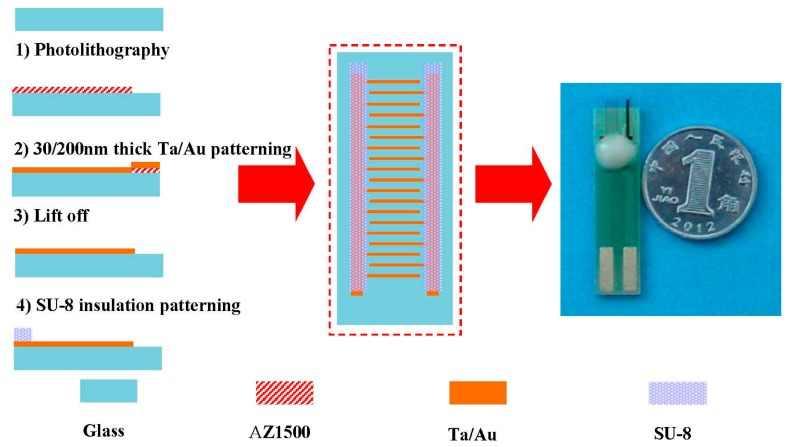
The fabrication process and a photograph of a gold MAE (coin diameter 19 mm).

The microelectrode chips were fabricated by the MEMS technique. The fabrication process and a photograph of the gold electrodes are shown in Figure 1. The MAE electrode is based on aninter-digitated electrode which consists of two sets of 50 fingers. Each finger is 10 μm in width, 2000 μm in length, and the gap between two adjacent fingers is 50 μm. A 3-inch diameter and 0.5 mm thick glass substrate was first coated with a positive photoresist. After development of the photoresist, metal films consisting of Ta (300 Å) and Au (2000 Å) were deposited by sequential sputtering. The Au layer is used as working electrode and the Ta layer is used as a seed layer as well as an electrical connection between glass substrate and Au layer. The photoresist and the films were removed from the unwanted areas in an acetone bath. After the lift-off process, SU-8 negative photoresist was coated as insulating layer. The microband array electrode was attached on PCB board for the electrical connection with the electrochemical work station. The modification of AuNPs was performed by the method of chronoamperometry at the potential of −0.3 V for 300 s [26]. Because the microband array electrode is the working electrode, the valid area of the microelectrode is 0.5 mm^2^.

### 2.3. Measurement Procedure

The microsensor measurements were carried out with a three-electrode-system which consists of a platinum plate as counter electrode, a gold MAE as working electrode, and an Ag/AgCl electrode as reference electrode in polypropylene bottles. To ensure the stability and consistency of the phosphate determinations, the three electrodes were fixed in a triangular shaped fixture. The distance between counter electrode and working electrode is 1 cm, the distance between counter electrode and reference electrode is 0.5 cm, and the distance between working electrode and reference electrode is 0.8 cm. The microsensor was first cleaned in 0.01 mol/L sulfuric acid (H_2_SO_4_) solution using cyclic voltammetry (CV) for ten cycles. The CV analyses were performed using applied voltages between 0 and 1.5V with a scan rate of 50 mV/s. The cleaned microsensor was immersed in the detection mixture solutions. The detection was carried out using linear sweep voltammetry from 0.5 V to −0.1 V. The recorded results were the reduction response currents at the applied votlage of −0.07 V *vs.* the Ag/AgCl reference electrode for different concentration samples.

## 3. Results and Discussion

### 3.1. Electrochemical Response Mechanism

Phosphate is not an electroactive substance, therefore the electrochemical detection of phosphate is based on the formation of a complex formed by its reaction with molybdate in an acidic solution to form a Keggin anion (PMo_12_O_40_^3−^) according to Reaction (1). In order to ensure that all the phosphates present are involved in the complexation reaction, the concentration of molybdate used in this work is 20 mM, which is in large excess over the reaction stoichiometry (∼10 μM) [20]. The pH is adjusted to 1.0 with sulfuric acid.

(1) $${{\text{PO}}_{4}}^{3-}+12{{\text{MoO}}_{4}}^{2-}+{27\text{H}}^{+}\to {\text{H}}_{3}{\text{PMo}}_{12}{\text{O}}_{40}+{12\text{H}}_{2}\text{O}$$

**Figure 2.**
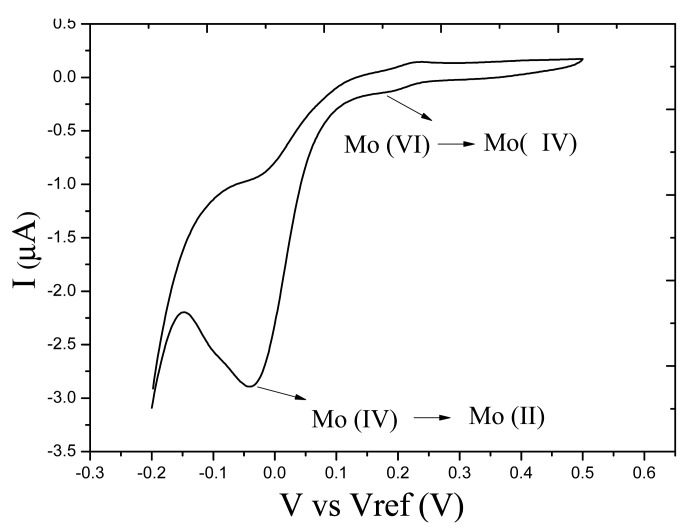
Cyclic voltammetric responses of molybdophosphate complex with an unmodified microelectrode.

For the CV responses of the molybdophosphate complex, there are two reduction peaks and two oxidation peaks in one cycle. The first reduction peak indicates that Mo (VI) is reduced to Mo (IV), and the second reduction peak is produced by the reduction from Mo (IV) to Mo (II). Figure 2 shows the cyclic voltammetric responses of the molybdophosphate complex with a concentration of 0.40 mg P/L. The peak current value of the reduction wave is related to the concentration of phosphate. Comparing the two reduction peaks, the second peak is much sharper than the first peak. Therefore, to avoid the influence of background current, the phosphate detection voltage was chosen as −0.07 V *vs.* Ag/AgCl reference electrode, which is at the second reduction peak.

### 3.2. Electrochemical Characterization of AuNPs-Modified Microelectrode

**Figure 3.**
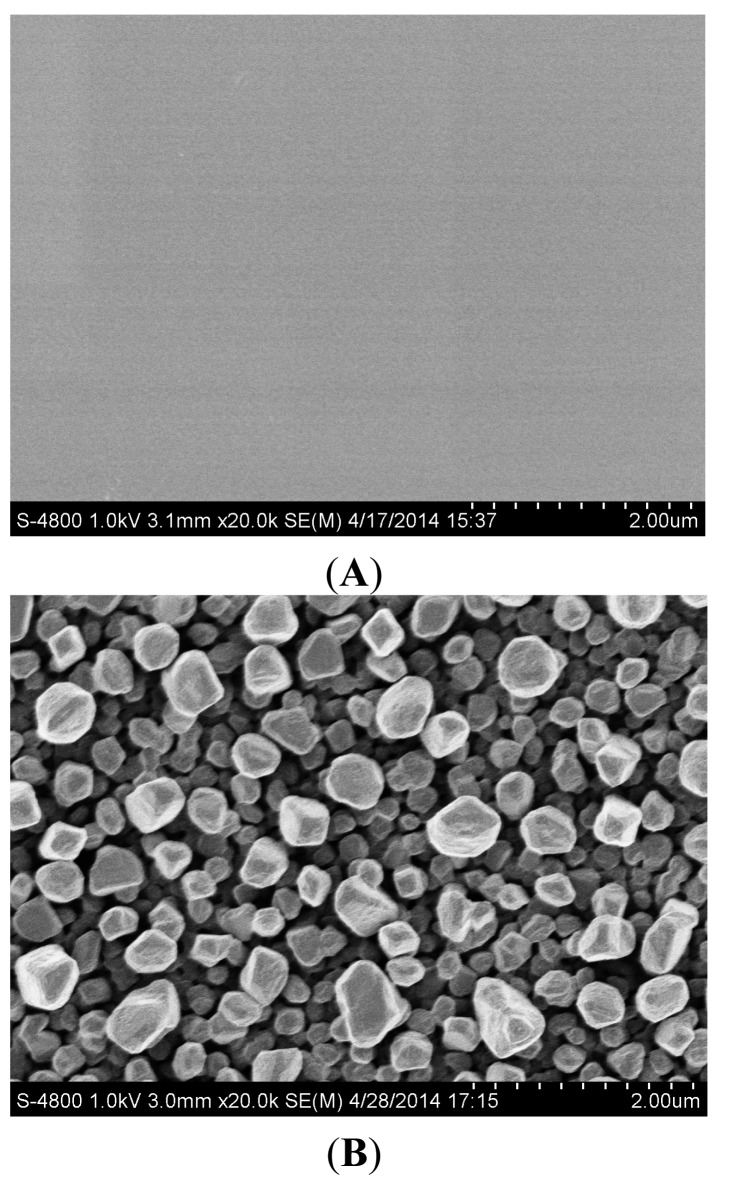
SEM images of bare (**A**) and AuNPs-modified MAE (**B**).

**Figure 4.**
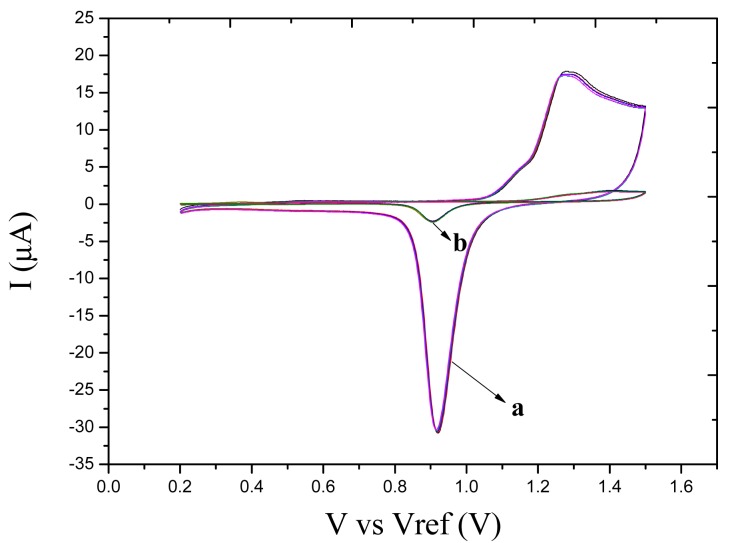
Cyclic voltammetric response in 0.01 M sulfuric acid. **a**: AuNPs-modified microelectrode; **b**: unmodified microelectrode.

The surface morphology of the electrode was investigated by scanning electronic microscope (SEM). The SEM images of MAE (Figure 3) revealed that the unmodified microelectrode (Figure 3A) showed at two-dimensional flat morphology while the AuNPs-modified electrode (Figure 3B) had a three-dimensional structure and the average diameter of AuNPs was about 500 nm. The cyclic voltammetry responses in 0.01M sulphuric acid are presented in Figure 4. The uniform reduction peaks show that the microsensor was cleaned well and in stable state. The reduction peak current of the modified electrode was 15 times that of the unmodified electrode. This difference results from the mechanism of growth of the three-dimensional crystals.

### 3.3. Electrochemical Response and Phosphate Determination

**Figure 5.**
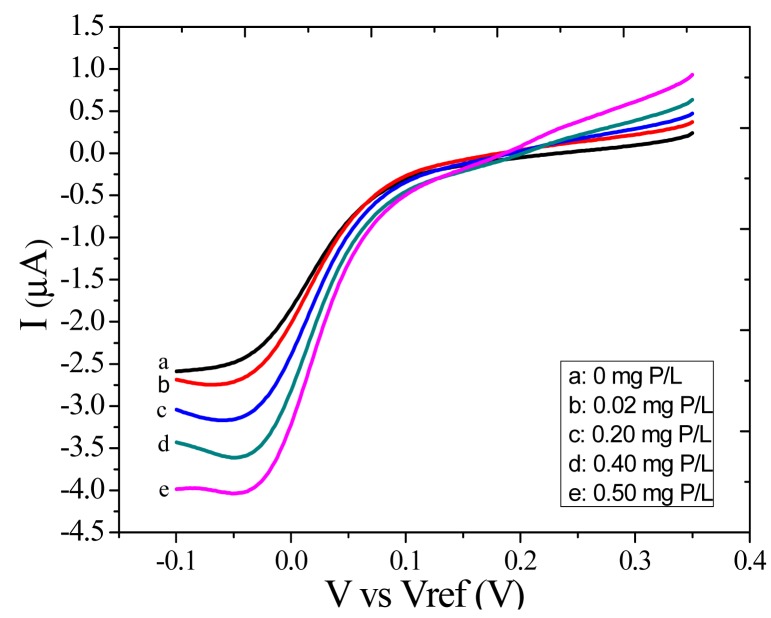
LSV responses of different concentrations of phosphate.

The determination of phosphate was carried out using linear sweep voltammetry from 0.5 V to −0.1 V. The LSV responses of phosphate were recorded with different concentrations from 0.02 to 0.50 mg P/L. The current responses of the unmodified microelectrode are shown in Figure 5. We can see that the response current decreases with the decrease of voltage and the second reduction peak currents decrease with the increase of phosphate concentration.

**Figure 6.**
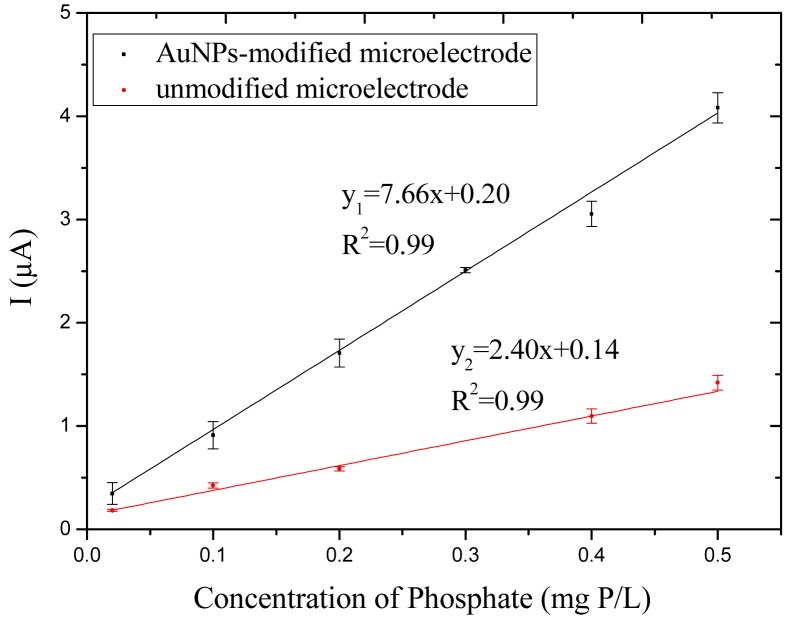
Calibration curve of phosphate standard solution measurement.

The calibration curve in Figure 6(1) shows that the unmodified phosphate microsensor has a linear response to phosphate with a sensitivity of 2.40 μA per (mg P/L) (*R*^2^ = 0.99) and the AuNPs-modified microsensor in Figure 6(2) has a sensitivity of 7.66 μA per (mg P/L) (*R*^2^ = 0.99). It is found that the sensitivity of the microelectrode was enhanced three times after the AuNPs modification. The main reason of this phenomenon may be that the AuNPs modification led to a larger area-to-volume on the surface of the gold microelectrode. The larger effective surface may contain a larger number of active sites, which results in an increase of the current density on the surface of the working area, and an improvement of the sensitivity of the microelectrode. The microsensor has a linear range of 0.02–0.50 mg P/L. The limit of detection (LOD) was calculated as 3 (SD/S), where SD is the standard deviation of the current response of a blank sample, and S is the slope of the calibration curve. The values of LOD using the unmodified electrode and the AuNPs modified electrode are calculated as 12.5 and 41.0 μg/L, respectively. Table 1 shows the differences between some different gold microsensors for phosphate determination. Compared with chronoamperometry for the detection of phosphate [21,22], this microsensor using LSV method has a much higher current response and less background current influence.

### 3.4. Total Phosphate Determination

To examine the performance of the unmodified gold microelectrode and AuNPs-modified microelectrode for TP determination, a real water sample from the Wanquan River at Tsinghua University was tested. The digestion of this sample was performed using a Lianhua 5B-6P digestion device (Lianhua Tech. Co., Ltd., Lanzhou, China). First, a real sample (8 mL) and oxidant (1 mL) (see Section 2.1) were mixed and sealed in a reaction tube. Then the tube was put into the digestion device and heated at 120 °C for 30 min. After digestion, the sample was cooled to room temperature in a cold-water bath. As a traditional standard method for TP detection, a spectrophotometric method was used to determine the concentration of the real sample. Then, the digested sample was mixed with 20 mM molybdate sodium and detected by the LSV method using the unmodified micro electrode and AuNPs-modified microelectrode, respectively. The measurement results of the real sample are listed in Table 2.

## 4. Conclusions/Outlook

In this work, an electrochemical microsensor based on an AuNPs-modified gold microband array electrode was developed for the determination of phosphate in freshwater samples. The working principle of this microsensor is based on the reduction of molybdophosphate complex in pH = 1.0. Under the potential of −70 mV *vs.* Ag/AgCl reference electrode, the second reduction peak of molybdophosphate complex offers different response currents for different phosphate concentrations. The AuNPs-modified microsensor has a linear response to phosphate with a linear range of 0.02 ∼ 0.50 mg P/L. The experimental results showed that AuNPs-modified microelectrode has better sensitivity and a larger current response than the corresponding unmodified microelectrode. In future studies, the sensitivity will be improved by changing the dimensions and structure of the microband array electrode. This method could be applied in the determination of TP in real water samples.

## Figures and Tables

**Table 1.** The current response sensitivities of different gold microsensor for phosphate detection.

**Electrode**	**Electrode Area (mm^2^)**	**Current Response Sensitivity (μA/mm^2^·** **(mg/L)^−1^)**	**Limit of Detection (μg/L)**
Unmodified gold electrode in this paper	0.5	4.80	12.5
AuNPs-modified gold electrode in this paper	0.5	15.32	41.0
Jońca [20]	7.065	0.0787	3.72
Bai [21]	0.45	0.0471	20.0
Bai [22]	0.45	0.0638	3.72

**Table 2.** The measurement results of a real sample.

**Spectrophotometric Method (mg P/L)**	**Unmodified Microelectrode**		**AuNPs-Modified Microelectrode**	
	
	**Measurement Result (mg P/L)**	**Recovery (%)**	**Measurement Result (mg P/L)**	**Recovery (%)**
0.105	0.110	104.76	0.131	124.76
	0.115	109.52	0.124	118.09
	0.114	108.57	0.111	105.71

average	0.113	107.62	0.122	116.19
